# Supplementary figures and images for: Cell autonomous role of leucine-rich repeat kinase in protection of dopaminergic neuron survival
Source: bioRxiv. 2024 Mar 7:2023.10.06.561293. Originally published 2023 Oct 10. Preprint. [Version 5] doi: 10.1101/2023.10.06.561293 (PMC10592668; doi:10.1101/2023.10.06.561293)

Figure 1-I  
LRRK1 WB  
control  
LRRK1 KO

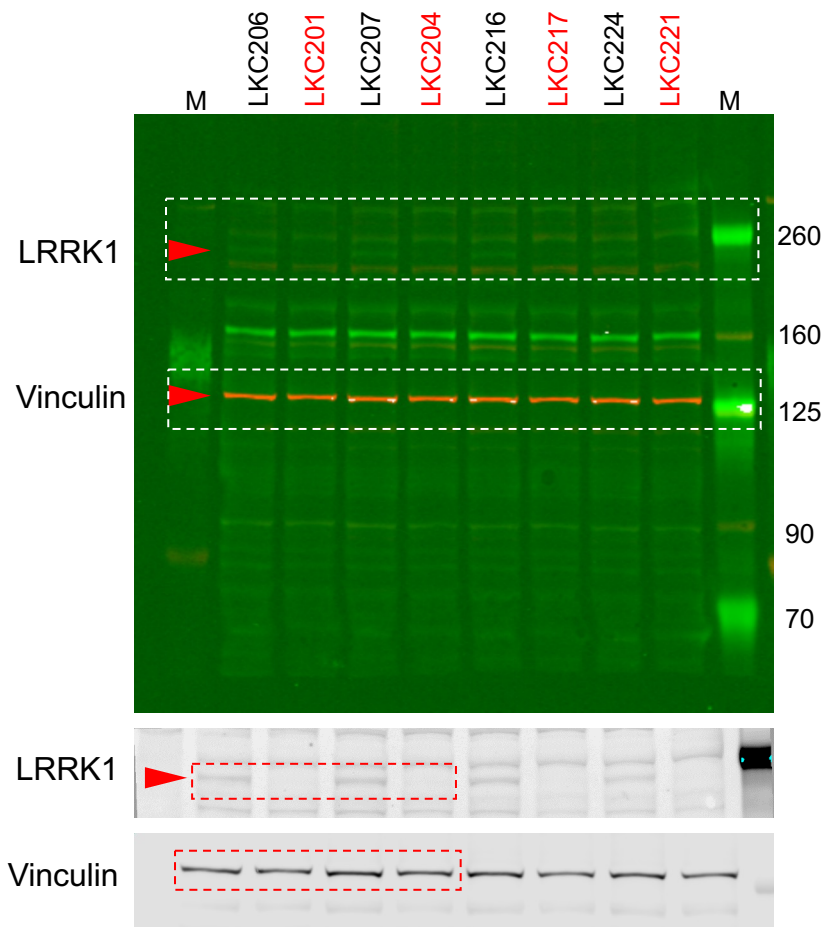

Figure 1-I  
LRRK2 WB  
control  
LRRK2 KO

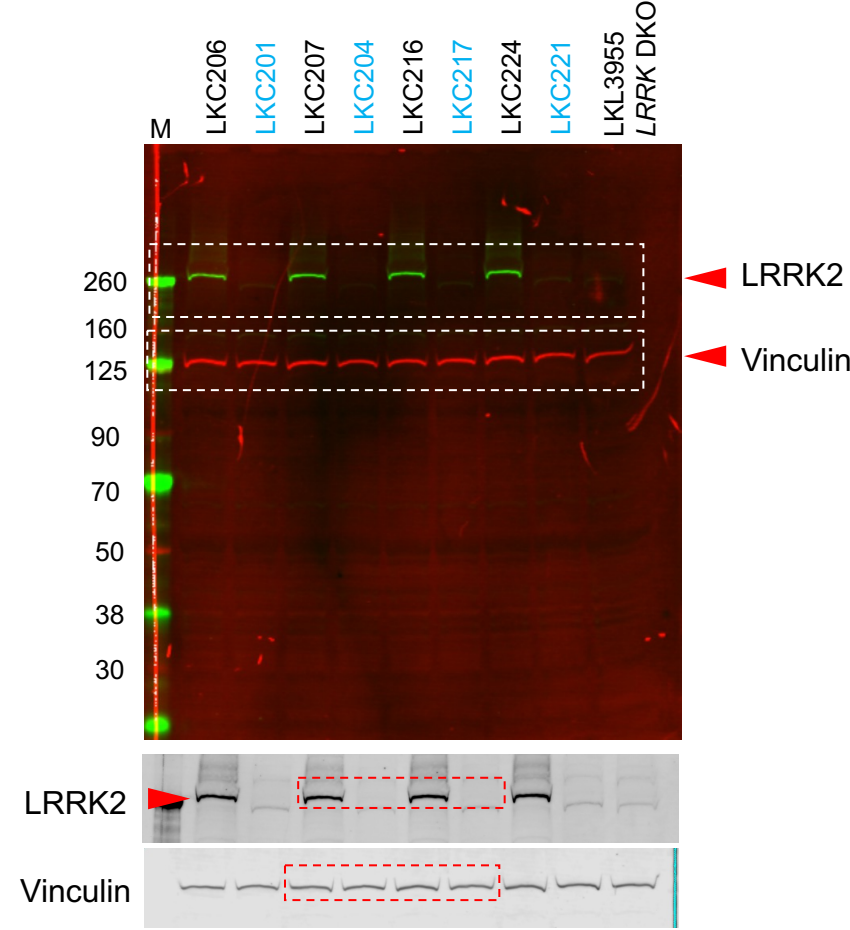

Supplement: Supplement 2 [file media-2.pdf]
